# Frequency of Positive Surgical Margin at Prostatectomy and Its Effect on Patient Outcome

**DOI:** 10.1155/2011/673021

**Published:** 2011-06-09

**Authors:** Kenneth A. Iczkowski, M. Scott Lucia

**Affiliations:** Department of Pathology, School of Medicine, University of Colorado Denver, Aurora, CO 80045, USA

## Abstract

A positive surgical margin at prostatectomy is defined as tumor cells touching the inked edge of the specimen. This finding is reported in 8.8% to 42% of cases (median about 20%) in various studies. It is one of the main determinants of eventual biochemical (PSA) failure, generally associated with a doubled or tripled risk of failure. The effect of a positive margin on outcome can be modified by stage or grade and the length, number and location of positive margins, as well as by technical operative approach and duration of operator experience. This paper tabulates data from the past decade of studies on margin status.

## 1. Introduction

### 1.1. Definition of a Positive Surgical Margin (PSM) in Radical Prostatectomy Specimens

**Figure 1:**
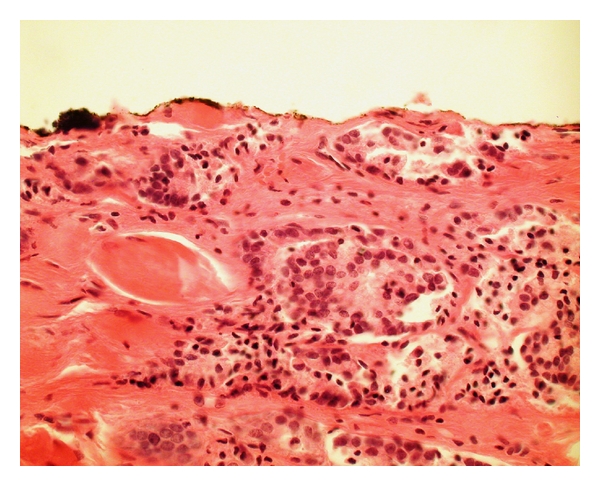
Prostatectomy specimen with a definite positive surgical margin (PSM). The inked resection margin transects tumor (400x).

As with all surgical specimens resected for cancer, the margins of a prostatectomy specimen are inked, usually using one color dye for the right side and one for the left. It is the pathologist's task to assess the microscopic slides and determine the proximity of tumor glands or cells to the ink to decide whether there is a definite positive surgical margin (PSM) (Figure 1).

**Figure 2:**
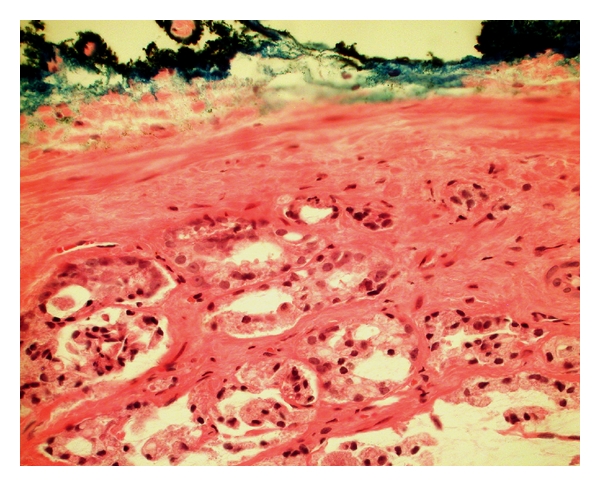
Prostatectomy specimen with negative surgical margin. Tumor approaches within less than 1 millimeter of the inked margin (400x).

A fundamental question is whether a tumor focus that is close to, but not touching, the resection margin (Figure 2) holds the same implications as a PSM. This question was first answered by Epstein and Sauvageot in 1997, in a study of 101 cases [1]. They found that patients with biochemical progression were no more likely to have tumor close to the margin than those without progression. Emerson et al., confining their study to just 278 margin-negative whole-mount prostate cases, validated that the closest distance between tumor and resection margin was not a significant predictor of PSA recurrence by univariate or multivariate analysis [2]. Thus, it was the consensus of the International Society of Urological Pathology in 2009 not to mention in written reports if tumor merely approaches but does not touch the margin [3]. This contrasts with the practice in other types of specimens such as breast lumpectomy specimens, in which the distance of tumor close to the margin is reported and does matter for outcome.

A PSM is a strong determinant of the probability of biochemical failure and is at least as important as grade, stage, and preoperative serum prostate-specific antigen (PSA). In unselected contemporary studies the PSM rate ranges from 8.8% [4] to 37% [5]. The interobserver reproducibility of designation of a PSM by urologic pathologists, using the definition of tumor on ink, has been shown to be good to excellent. The kappa value is 0.73 for definitive surgical margin status [6]. This supports the validity of many studies in concluding that, compared to negative surgical margin (NSM) status, a PSM correlates with a significant rise in biochemical failure rate. The purpose of this paper is to provide a compendium for urologists and their patients of all that is known about prostate margin status as an outcome predictor.

## 2. Methods

A review of papers pertaining to prostate margin status and its effect on outcome was undertaken using PubMed searches from 1997 to the present.

## 3. Results

### 3.1. Can Prostate Biopsy Results Predict Margin Status?

We undertook a study a few years ago to determine the extent to which prostate biopsy results could predict cancer at prostatectomy that is unifocal, unilateral, margin-negative, and of small volume [20]. These four factors are the main criteria for choosing minimally invasive therapies such as targeted focal ablation of the prostate, as alternatives to radical prostatectomy. Unilateral cancer at prostatectomy was predicted by unilateral cancer in the biopsy (OR, 4.30) and unifocal cancer in the biopsy (OR, 2.63). In that study, negative surgical margins were predicted by unilateral cancer in the biopsy (OR 2.53, positive predictive value 82%). Therefore, biopsy findings can strongly predict prostatectomy margin status and other findings.

### 3.2. Comparison of PSM Rates by Technical Approach (Table 1)

In the past decade, nonrobotic or robotic laparoscopic techniques have been increasingly used in place of conventional open radical prostatectomy. The laparoscopic approaches are often considered superior for continence and potency [8, 11, 12, 14, 16]. Most studies involving prostate pathology after laparoscopic approaches have found a PSM rate comparable with that of an open approach [7, 8, 14, 15, 19]. PSM rates were as follow: open, 7.6% [10] to 41.6% [18]; laparoscopic without robot, 11.3% [19] to 21.3% [8]; robotic, 13% [15] to 24.44% [18].

PSM rate for robotic approaches was found to be significantly worse than that for open ones (*P* = .007) in one study [10]; however, two other studies found open approaches superior to the robotic ones [17, 18]. In the study that found the open approach better, the result was confounded by nerve sparing, so robotic prostatectomies showed a nonsignificant trend toward lower PSM for a non-nerve-sparing approach (*P* = .09) [10]. When the anterograde open approach was compared with the retrograde approach, significantly fewer PSMs were found by retrograde approach (*P* = .03) [9]. 

In a comparison of robotic versus nonrobotic laparoscopic approaches, one study found the robotic method superior [8]. Another found that the outcome was highly stage dependent, with 7% of pT2 patients with biochemical failure as opposed to 34% of pT3 patients [11]. Failure could also depend on number of positive margins [16]. In a study evaluating the robotic approach, a lower PSM rate was achieved by cold incision of the dorsal venous complex before suture ligation [12]. 

### 3.3. Comparison of PSM Rates by Duration of Surgical Experience (Table 2)

In the above comparison of surgical approaches, it must be noted that the new laparoscopic approaches have a demonstrable learning curve. That is, in three studies conducted in the middle of the 2000–2010 decade, the PSM rate improved after a few years of practice [21–23]. While a significant decrease in PSM rate occurred over time with a laparoscopic approach, PSM held steady for open procedures during the same time period [19]. Even with the open approach, during the 1990s and early 2000s, one study had noted that there was also a learning curve with respect to the PSM rate [24].

It is a bit disconcerting but it also must be admitted that individual surgeons may vary in their frequency of PSMs. In a study of 4,629 men operated on by open prostatectomy by one of 44 surgeons, for the 26 surgeons who each treated >10 patients, the rate of PSM ranged from 10% to 48% [33]. A 6-fold difference was even reported for the same surgeon at different institutions [13].

### 3.4. Margin Status Effect on PSA Failure Rate at 10 Years (Table 3)

PSM rates in studies not comparing approaches ranged from 13% [25] to 42% [31] with a median 23% [27]. In the presence of a PSM, the failure rate was either double [28, 30, 32, 34, 40, 42, 43], triple [5, 26, 29, 38] or showed an increase of greater magnitude [4, 39] compared to NSM. Two studies did not specify this [5, 30]. In studies reporting a Hazard Ratio (HR) comparing a PSM to NSM, the HR ranged from 1.3 [46] up to 3.66 [42].

### 3.5. Tumor Stage (Table 4) or Grade (Table 5) Can Modify the Effect of PSM on PSA Failure Rates, at 10 Years

Nine studies compared PSA failure rates as a function of pathologic stage pT3a and pT3b versus pT2 or of pT3 versus pT2. (The apparent stage sometimes cannot be assessed because of capsular incision [58].) Failure rates with a PSM in stage pT2 ranged from 10.6% [38] to 63% [42], with an HR of 1.7 [4] to 3.81 [34] compared to having an NSM. For stage pT3a, failure rates were 38% [35] to 58% [36], with HR ranging from 1.4 [46] to 3.6 [4] compared to NSM. For stage pT3b, one study reports 71% failure, with HR of 1.4 compared to NSM [35]. Some studies chose to combine both pT3 substages and disclosed failure rates from 57% [37] to 75% [43] and HR of 4.1 [37] to 11.85 [38]. Thus, PSM exerts an effect that is synergistic with increasing stage, although the HR compared to NSM seems fairly constant across stages pT2, pT3a, and pT3b, at about 3 to 4. A study examining the phenomenon of capsular incision, sometimes denoted pT2+, found a 29.3% failure rate versus 7.3% for no incision (*P* < .0001) [46].

The HR for failure with a PSM seems to increase with increasing Gleason score [4, 35, 42, 44]. In one study [34], however, after controlling for Gleason score, a PSM versus NSM with Gleason ≤7 was significantly predictive of failure, while PSM versus NSM with Gleason ≥8 was not (*P* = .115). Finally, Cao et al. noted that the Gleason score at the positive margin was predictive of biochemical recurrence [59]. Also, as the Gleason score of the main tumor rose, the concordance with the grade at the margin diminished: 99% for score 6 but 38% for score 9. By multivariate analysis, Gleason score at the margin predicted biochemical failure (*P* < .05) [59].

### 3.6. The Effect of PSM on Mortality Rate at 10 Years Is Also Modified by Stage and Grade (Table 6)

Three studies addressed the prostate cancer-specific death rate in the presence of a PSM. Two studies, one based on the SEER cancer data registry [45], found a significantly higher death rate at 10 years in the presence of a PSM [34, 45], namely, 0.86% versus 0.33%  (*P* < .001) and 2.6% versus 0.6% which was significant (*P* = .006). In another study, from the Mayo Clinic registry, a PSM was not a significant predictor of death among 11,729 cases (*P* = .15), but did predict death in the subset that was stage pT3 [34].

### 3.7. PSA Failure Rates after a PSM Are Influenced by Length and Number of PSM (Table 7) and by Location of PSM (Table 8)

Many pathologists report the length of a PSM. Using categorical PSM length cut-offs between 3 mm and 10 mm, length significantly affected outcome in many [36, 41, 47–49, 58] but not all [50–52] studies. Emerson et al. [53] found a PSM length >3 mm to be a significant outcome predictor by univariate analysis but it fell short of significance by multivariate analysis (*P* = .076) [53]. Moreover, the length of PSM by frozen section predicted residual tumor in additionally resected neurovascular bundles by multivariate analysis (*P* < .001) [55].

The number of PSMs probably lacks predictive value. In most studies, number of PSM was not significant for outcome [29, 31, 47, 49]. In two studies, multiple PSMs as opposed to a single PSM predicted failure (HR 1.4, *P* = .002 by multivariate analysis or HR = 2.19) [54, 58]. In another study, number of PSMs carried only borderline significance when ≥3 foci were positive compared to one (*P* = .06) and not significant for 2 foci compared to one [50]. Emerson et al. found that PSM number predicted failure by univariate analysis (*P* = .037) but lost most of its predictive value when adjusted for Gleason score (*P* = .076) [53]. 

The most common location of a PSM was in the posterior or posterolateral prostate [41, 47, 49], although one study found PSM equally common at the apex [24]. A positive apical soft tissue margin appears more consequential than a prostatic tissue margin [56]. Eastham et al. noted that the elevated risk of a posterior PSM means that “efforts to maintain adequate tissue covering including the routine excision of Denonvilliers' fascia and a component of the fat of the anterior rectal wall should be made in all patients…” [24]. Broken down by various sites, a posterolateral PSM predicted failure in most studies [24, 48] but not all [49]. 

Comparing various sites of PSM, the effect of an apical PSM was not significantly different from PSM at posterolateral or other sites [29, 52, 58], and another study concluded that the PSM location seemed not to predict failure [53]. However, in two studies, a positive posterolateral margin predicted failure while the apical margin did not [24, 57]. Possibly, residual apical tumor is less viable than residual tumor in the posterolateral region.

## 4. Conclusion

Prostate margin status is an important determinant of patient outcome after radical prostatectomy. In a 2010 College of American Pathologists survey, this feature was missing from 1% of pathology reports [60], thus the inclusion of this and other essential features is a quality assurance concern for pathologists. Most urologic pathologists endorse the reporting of the extensiveness of positive margins, expressed as length, number, or radial extent positive for tumor cells; all these measurements have some relevance toward outcome. The presence of a positive margin confers a 2-3-fold increased hazard ratio for biochemical recurrence—modified by stage and tumor grade—and necessitates close clinical followup.

## Figures and Tables

**Table 1:** Comparison of PSM rates by technical approach.

First author, yr	No. of pts	Cohort years	Median f/u, yr	Open		Laparoscopic		Robotic		Failure rate if PSM
				PSM rate	*P* value	PSM rate	HR, *P* val.	PSM rate	HR, *P* val.	
Williams 2010 [7]	4240	2004–2006		20.1%		17.4%		17.4%		
Coelho 2010 [8]	≥250^††^	1994–2009		24.0%		21.3%		13.6%		
Sciarra 2010 [9]	200	2003–2007		18% anterograde, 14% retrograde	*P* = .03	—		—		—
Williams 2010 [10]	950	2005–2008		7.6%		13.5%,	HR 1.9*, *P* = .007	—		—
Coelho 2010 [11]	876	2008-2009		—		—		pT2, 6.8%, pT3, 34.0%	*P* < .0001	—
Guru 2009 [12]	480	2005–2008		—		—		5% apical, 2% versus 8%**		—
Bong 2009 [13]	301	1994–2006	2.0	24.7% at 1 institution but 4.2% at another	*P* < .01***	—		—		25.6% at 1 institution but 100% at other
Hakimi 2009 [14]	150	2001–2008				13.7%		12%		6.7% versus 5.3% *P* = .37
Laurila 2009 [15]	192	2006		14%		—		13%	*P* = .5, no diff in apical margin	—
Terakawa 2008 [16]	137	2000–2007		PSM	Not signif.	—		More multiple PSM, get #		—
Smith 2007 [17]	400	2002–2006		35%^†^		—		15%	*P* < .001	—
Silva 2007 [18]	179	1999–2003		41.6%		—		24.44%	*P* = .023	—
Touijer 2007 [19]	1177	2003–2005		11.0%; pT2 5.3%, pT3 22.0%		11.3%; pT2 8.2%; pT3 17.2%	HR 1.2, *P* = .5	—		—

*OR falls to 1.6 if nerve-sparing is eliminated as a variable (*P* = .05).

**Lower rate achieved by cold incision of the dorsal venous complex before suture ligation.

***For the same surgeon; but higher average pathologic stage at the first institution.

^†^But open method was used for more high-risk cases and also cases with a higher preoperative PSA, *P* = .002.

^††^Review of several papers.

**Table 2:** Comparison of PSM rates by duration of surgical experience.

First author, yr	Number of cases	Cohort years	PSM rate		
			Open	Laparoscopic	Robotic
Rodriguez 2010 [21]	400, by intervals of 100	2004–2006	—	For pT2: 28.4%–31.9% to 11.6%–11.5%*	—
Yee 2009 [22]	50, then 250	2005–2008	—	—	Cases 1–50: 36%, 51–250: 17.6%, 251–450: 7.5%
Liss 2008 [23]	216	2003–2007	—	—	14.8%, decr. over time *P* = .03, nerve-sparing increased risk *P* = .03
Eastham 2007 [24]	2442	1983–1990 and 1991–2004	18% versus 10%, *P* = .001	—	—
Touijer 2007 [19]	1177	2003–2005	No decrease over time	Decreased over time, *P* = .0002	—

*First 200 cases versus last 200 cases.

**Table 3:** The effect of margin status on PSA failure rate at 10 years.

First author, yr	*n*	Cohort years	PSA fail criterion, ng/mL	% PSM, overall	% biochemical failure rate		
					PSM	NSM	*P* value, HR
Williams 2011 [25]	158^††^	2005–2009	—	13	No f/u		
Ahyai 2010 [26]	932	1992–2004	≥0.1	12.9	21.7	6.9	*P* = .001
Tsao 2009 [27]	100*	2004–2007	≥0.2	23	—		
Sæther 2008 [28]	219	1996–2004	≥0.2	32.4	40	18	*P* = .017
Pfitzenmaier 2008 [29]	406	1990–2006	≥0.2	17.2	64.3	20.5	*P* < .001, HR 3.21
Swanson 2007 [30]	719	1985–1995	≥0.3	15.3	63	27	*P* < .0001
Ahyai 2010 [26]	936	1992–2003	≥0.4	37	19	7	*P* < .01
Kausik 2002 [31]	1202^†^	1987–1995	>0.2	42	35	24	*P* = .0001
Menon 2010 [32]	1384	2001–2005*	≥0.2	25.1	—	—	*P* < .0001, HR 2.43 (1.72–3.42)

*Robotic only.

^†^pT3 cases only.

^††^pT2 cases only.

**Table 4:** Modification of PSA failure rates according to stage, at 10 years (unless specified).

First author, yr	*n*	Cohort Years	PSA fail criterion ng/mL	% PSM, overall	% biochemical failure rate			% biochemical failure rate with PSM by stage					
					PSM	NSM	*P* value HR	pT2	*P* value, HR	pT3a	*P* value, HR	Stage pT3b	*P* value, HR
Williams 2010 [7]	4240	2004–2006	—	19.4	No f/u			14.9		42		—	
Ploussard 2010 [34]	1943	2000–2008	>0.2	25.6	54.2	29.9	*P* < .001, HR 2.6		*P* < .001, HR 3.81		*P* = .001, HR 2.09		*P* = .1, HR 1.46
Budäus 2010 [35]	4490	1992–2008	≥0.1	18.9	—			17 versus 5	HR 2.9	38 versus 26	HR 1.9	71 versus 53,	HR 1.4
Brimo 2010 [36]	108^†^	1995–2008	≥0.2	Inclusion criterion^†^	—				—	58		—	
Hsu 2010 [37]	164	1977–2004	≥0.2	48.2 (all cT3)	—				—	57%, HR 4.1, *P* = .03			
Ficarra 2009 [38]	322*	2005–2008	≥0.2	29.5	6.2	1.8	*P* < .001 (at 12 mo.)	10.6		57.5	*P* < .001, HR = 11.8	72.2	
Kwak 2010 [39]	266	1995–2007	≥0.2	18.5	52.6	8	*P* < .0001	29.3 versus 7.3^$^	*P* < .0001	51 versus 10.5	*P* = .04 HR 1.4,	—	
Hashimoto 2008 [40]	238**	1985–2005	≥0.2	34.4	38.4	19.3	*P* < .001		HR = 1		*P* = .033 HR 3.36,		*P* = .002, HR 7.13,
Chuang 2007 [41]	135**	1993–2004	≥0.2	—	—			28.7 versus 3.3	*P* < .0001	Focal EPE 21.4% versus 10.3%, *P* = .02,			
										Ext EPE 41.5% versus 26%, *P* < .0001			
Orvieto 2006 [4]	996	1994–2004	≥0.1	8.8 (all); pT2 1.7, pT3a 24.9, pT3b 27.1	35	7.8	*P* < .001, HR 3.27		*P* < .001, HR = 1.7		*P* = .011, HR 3.6		*P* = .19, HR 6.5
Karakiewicz 2005 [42]	5831	1983–2000	≥0.1–≥0.4	26.7	63.9	29.9	*P* = .001, HR 3.66	63 versus 30	*P* < .001	—		—	
Swindle 2005 [43]	1369	1983–2000	≥0.4	12.9 (all); pT2 6.8, pT3 23	42	19	*P* = .002, HR 1.52	38.6 versus 19.6	*P* < .001	74.9% versus 53.8%, *P* < .001			

*Robotic only.

**Study used 5-year biochemical recurrence.

^†^Restricted to GS = 7, stage pT3a, and PSM.

^$^If there is capsular incision, versus no capsular incision.

**Table 5:** Modification of PSA failure rates according to grade, at 10 years (unless specified).

First author, yr	*n*	Cohort years	PSA Fail criterion, ng/mL	% PSM, overall	% biochemical failure rate			Gleason score effect on failure if PSM	
					PSM	NSM	*P* value, HR	Comparisons	*P* value, HR
Ploussard 2010 [34]	1943	2000–2008	>0.2	25.6	54.2	29.9	*P* < .001 HR 2.6	≤7 versus ≥8	*P* < .001 *P* = .115
Budäus 2010 [35]	4490	1992–2008	≥0.1	18.9	—	—		compared to GS = 6: for 3 + 4, for 4 + 3, for ≥8,	HR 2.81 HR 6.57 HR 9.86, all *P* < .001
Brimo 2010 [36]	108^†^	1995–2008	≥0.2	Inclusion criterion^†^	—	—		Score at margin	*P* = .007
Alkhateeb 2010 [44]	11,729^‡^	1992–2008	≥0.4	31.1	56	77	*P* < .0001 HR 1.63	Low risk 5.1% versus 0.4%; med. risk 17% versus 65%; hi. risk 43.9% versus 21.5%	—
Orvieto 2006 [4]	996	1994–2004	≥0.1	All 8.8; pT2 1.7, pT3a 24.9, pT3b 27.1	35	7.8	*P* < .001 HR 3.27	7 versus ≥8,	*P* < .001, HR 7.2 *P* < .001, HR 21
Karakiewicz 2005 [42]	5831	1983–2000	≥0.1 to ≥0.4	26.7	63.9	29.9	*P* = .001 HR 3.66	≥7	*P* ≤ .008, HR 2.81

^†^Restricted to GS = 7, stage pT3a, and PSM.

^‡^Risk groups based on Gleason score and preoperative PSA: low = PSA <10, Gleason ≤6; medium = PSA 10–20 or Gleason 7; high = PSA >20 or Gleason ≥8.

**Table 6:** Modification of prostate cancer mortality rates according to stage or grade, at 10 years.

First author, yr	*n*	PSA Fail criterion, ng/mL	PSM, %	Median f/u, yr	PCa death rate if			PSM rate or HR by stage		PSM rate by grade	
					PSM, %	NSM, %	*P* value, HR	pT2	pT3 a-b	Gleason ≥7	*P* value
Wright 2010 [45]	65,633	—	21.2	7	0.86	0.33	*P* < .001	17.7%	43.8%, *P* < .001	27.5% versus 18.3%	*P* < .001
Boorjian 2010 [34]	11,729	≥0.4	31.1	8.2	4	1	*P* = .15	HR 1.0	HR 2.1, *P* < .0001	—	—
Ploussard 2010 [34]	1943	>0.2	25.6	6.7	2.6	0.6	*P* = .006, 3.7 (1.5–9.5)	16.0	33.6–40.2	—	—

**Table 7:** Modification of PSA failure rates according to PSM length or number of PSM, at 10 years (unless specified).

First author, yr	*n*	Cohort years	PSA fail criterion ng/mL	Median f/u, yr	PSM, overall	%Biochemical failure rate			According to length at margin		According to number of PSM	
						PSM	NSM	*P* value, HR	Fail rate with PSM	HR and *P* value	Fail rate with PSM	HR and *P* value
Brimo 2010 [36]	108^†^	1995–2008	≥0.2	3.0	Inclusion criterion	—	—		>3 mm: as continuous variable	*P* = .004 *P* = .015	—	—
van Oort 2010 [47]	174*	1995–2005	≥0.1	3.0	Inclusion criterion	29	—		>10 mm, 39% versus 21%	HR 2.3, *P* = .022	>1 versus 1	HR 1.46 *P* = .24
Lake 2010 [48]	1997	1996–2008	>0.2	4.1	18, 6.7 for T2	ext. 62, focal 36	16%	*P* < .0001	extensive 62%, focal 36% negative 16%	*P* < .0001	—	—
Stephenson 2009 [46]	7160	1995–2006	≥0.2	3.2	21	40		*P* < .001, HR = 2.3	extensive 66%, focal 34%	HR 1.3, *P* = .004^†^	multiple 83%, one 17%	HR 1.4, MVA ^†^ *P* = .002
Shikanov 2009 [49]	1398	2003–2008	≥0.1	1.0	17**	—		*P* < .0001, HR 4.4	<1 mm 1–3 mm >3 mm	HR 0.26 HR 9.6, *P* = .03 HR 14.8, *P* = .01	?	*P* = .3 for fail
Goetzl 2009 [50]	103	1998–2008	≥0.2	—	23.3	—	—		≥6 mm	HR 1.7, *P* = .10	≥3 PSM versus 1 versus 2 PSM	HR 1.3, *P* = .06 Not sig.
Pfitzenmaier 2008 [29]	406	1990–2006	≥0.2	5.2	17.2	64.3	20.5	*P* < .001, HR 3.21	—	—	≥3 versus 1	*P* = .46
Marks 2007 [51]	158	1990–1998	≥0.1	4	—	55	—		≥5 mm	HR 1.00, *P* = .26	—	—
Vis 2006 [52]	281	1994–1999	≥0.1	6.75	23.5	33.3	7.9	*P* < .005	Focal versus extensive	*P* = .49	—	—
Emerson 2005 [53]	369	1999–2003	≥0.1	1.0	23	25.6	—		Median 3 mm	*P* = .031 univariate but .076 multivar.^††^	Mean 2.45 versus 1.80	*P* = .037 by univar. analysis
Sofer 2002 [54]	498		≥0.2	4 yr 5 mo	19.7			HR 2.8, *P* < .05			≥2, versus 1	*P* = NS
Kausik 2002 [31]	1202^†††^	1987–1995	>0.2	4.9	42	35	24	*P* = .0001	—		≥2, 62% versus 1, 65%	*P* = NS
Fromont 2004 [55]	734	1992–1999	≥0.2		25	—			—		>2 versus 1	HR 2.19, *P* not done

*Study used 5-year biochemical recurrence.

**Robotic only.

^†^But a predictive model nomogram does not improve accuracy of predicting failure after prostatectomy.

^††^Linear extent of positivity was associated with other pathologic variables such as preoperative PSA and tumor volume and not independently predictive when adjusted for Gleason score.

^†††^pT3 cases only.

**Table 8:** Location of PSM and their modification of PSA failure rates, at 10 years (unless specified).

First author, yr	*n*	Cohort years	PSA fail criterion, ng/mL	Medi-an f/u, yr	%PSM, overall	%Biochemical failure rate			Failure according to PSM location		Most common location
						PSM	NSM	*P* value, HR	% fail:	HR and *P* value	
van Oort 2010 [47]	174***	1995–2005	≥0.1	3.0	Inclusion criterion	29			—		Post 43%, ant 35%, apex 33%
Lake 2010 [48]	1997	1996–2008	>0.2	4.1	18, 6.7 for T2	Ext. 62 focal 36	16	*P* < .0001	Apex Ant Posterolat	HR 2.24, *P* = .03, HR 3.7, *P* < .0001 HR 2.5, *P* = .002	—
Godoy 2009 [56]	246^∗∗, ∗∗∗^	2000–2006	>0.15	2.8	Apical surgical, 3.2, apical soft tissue, 6.6; total 9.8	—			Apical surgical 48.6%, apical soft tissue, 4.7%***		
Stephenson 2009 [46]	7160	1995–2006	≥0.2	3.2	21	40	HR = 2.3	*P* < .001	Apex versus other	HR 1.1, *P* = .3	—
Shikanov 2009 [49]	1398	2003–2008	≥0.1	1.0	17**	—	—	*P* < .0001 HR 4.4	Posterolateral	*P* = .7 for fail	Posterolat 45%; apex 29%; base 6%
Pfitzenmaier 2008 [29]	406	1990–2006	≥0.2	5.2	17.2	64.3	20.5	*P* < .001 HR 3.21	Apex versus nonapex	*P* = .21	
Eastham 2007 [24]	2442	1983–2004	≥0.2	2.9	11.2, pT2 7, pT3 22	25	10	*P* = .0005 HR 1.39	Posterolat. Posterior	HR 2.80 HR 1.96 versus neg, *P* < .0005	Apex 37%, posterolat 35%
Chuang 2007 [41]	135^†∗∗∗^	1993–2004	≥0.2	—	—	28.7***	3.3	*P* < .0001			posterolat 61.5% post 19% ant 9%
Vis 2006 [52]	281	1994–1999	≥0.1	6.75	23.5	33.3	7.9	*P* < .005	Apex versus other	*P* = .65	—
Emerson 2005 [53]	369	1999–2003	≥0.1	1.0	23	25.6	—		Location, gen'l:Ass'n for # of lateral sites:	*P* = .437 *P* = .06	
Pettus 2004 [57]	498		≥0.2	4.4	19.7			HR 2.9, *P* < .05, See breakdown	apex 21% nonapex 26%	*P* = .25, HR 2.25, *P* < .05, HR 2.96	apex 5.6, nonapex 11.4
Kausik 2002 [31]	1202^††^	1987–1995	>0.2	4.9	42	35	24	*P* = .0001	—		apex 46% post. 64%
Sofer 2002 [54]	734	1992–1999	≥0.2		25	—	—	—	—		apex 45%; post. 32%

**Robotic only.

***Study used 5-year biochemical recurrence.

^†^pT2 cases only.

^††^pT3 cases only.
